# Do surrounding figures' emotions affect judgment of the target figure's emotion? Comparing the eye-movement patterns of European Canadians, Asian Canadians, Asian international students, and Japanese

**DOI:** 10.3389/fnint.2012.00072

**Published:** 2012-09-27

**Authors:** Takahiko Masuda, Huaitang Wang, Keiko Ishii, Kenichi Ito

**Affiliations:** ^1^University of AlbertaEdmonton, AB, Canada; ^2^Kobe UniversityKobe, Japan; ^3^Institute on Asian Consumer Insight, Nanyang Technological UniversitySingapore

**Keywords:** Asian Canadians, Asian international students, cognition, culture, emotion, European Canadians, Japanese, the affective context effect

## Abstract

Although the effect of context on cognition is observable across cultures, preliminary findings suggest that when asked to judge the emotion of a target model's facial expression, East Asians are more likely than their North American counterparts to be influenced by the facial expressions of surrounding others (Masuda et al., 2008b). Cultural psychologists discuss this cultural variation in affective emotional context under the rubric of holistic vs. analytic thought, independent vs. interdependent self-construals, and socially disengaged vs. socially engaged emotion (e.g., Mesquita and Markus, 2004). We demonstrate that this effect is generalizable even when (1) photos of real facial emotions are used, (2) the saliency of the target model's emotion is attenuated, and (3) a specific amount of observation time is allocated. We further demonstrate that the experience plays an important role in producing cultural variations in the affective context effect on cognition.

Previous studies suggest that East Asians tend to holistically pay attention to both focal and contextual information, while North Americans are more likely to pay attention to the focal objects (Nisbett et al., 2001; Nisbett, 2003; Nisbett and Masuda, 2003; Nisbett and Miyamoto, 2005). Under the rubric of analytic vs. holistic thought, Nisbett and his colleagues speculated that North Americans and East Asians epistemologically apply different strategies to the viewing of scenes. Researchers have in fact demonstrated systematic cultural variations in attentional patterns. For example, East Asians are more likely than their North American counterparts to describe contextual and relational information and to remember objects in relation to context (Masuda and Nisbett, 2001); to be good at finding the change in the spot-the-difference task (Masuda and Nisbett, 2006; Miyamoto et al., 2006); to perform well on a task that requires attention to context (Kitayama et al., 2003); and to perform less well on a task that requires attention to focal objects (Ji et al., 2000; Masuda et al., 2008a).

However, the effect of context on Europeans' and North Americans' cognitive judgment has also been reported. Researchers have shown that Western participants' judgment of target facial emotions is influenced by descriptions of concrete situations in which a particular facial emotion occurred (Russell and Fehr, 1987; Russell, 1991; Carroll and Russell, 1996). For example, Carroll and Russell (1996) demonstrated that Canadian participants' categorization of facial emotion was influenced by situational information. In their experiment, participants were sequentially presented with the description of a situation, and then a target facial emotion. In this paradigm, the contextual information was mismatched with the target models' facial expression. That is, anger-provoking situations (e.g., having a hubcap stolen from their car) were paired with fearful faces; fear-provoking situations (e.g., bumping into a bear while hiking) were paired with angry faces; and disgust-provoking situations (e.g., finding a rotten garbage bag in a kitchen after a long trip) were paired with sad faces. Participants then engaged in the judgment task. They were asked to select from a list of emotion words that best described the target model's feeling (happiness, anger, sadness, surprise, fear, or disgust). The results indicated that 70% of participants were influenced by the emotional valence of the situations, rather than the actual facial expression. In general, these findings suggest that Westerners treat the salient context information, rather than the facial expression, as the dominant element when judging the target's facial emotion. According to James Russell, a leading researcher on the effect of context on facial emotion judgment, “judgment of emotion in a facial expression is not a simple straightforward registration of the meaning of that face. The face is judged not in an absolute manner but relative to the context of judgment” (Russell, 1991, p. 150).

The literature of the affective priming paradigm supports the findings of the context effect, indicating that affectively salient contextual information influences people's judgment. For example, in a typical affective priming experiment, participants are briefly presented with various affectively salient stimuli (e.g., a beach under a clear blue sky, or a ruined building), and then categorize positive, neutral, or negative target stimuli (see Fazio, 2001, for a review). Participants' judgment is influenced by affective contexts in the prime, to the extent that the participants tend to respond faster to the target when the prime and the target are affectively congruent than when the two are incongruent. Several studies suggest that such an affective context effect is observable even when the contextual information is conveyed through words (Fazio et al., 1986); images of landscape scenery (Hietanen et al., 2007); nonverbal sounds (Carroll and Young, 2005); odors (Leppänen and Hietanen, 2003); attractive faces (Olson and Marshuetz, 2005); humorous cartoons (Strick et al., 2009); or recollection of outrageous events (Baumann and DeSteno, 2010). However, the extent to which people's cognition (judgment of the target face) is influenced by the affective context, and whether there are systematic cultural variations in the degree of context effect, have not been fully examined.

Extending the theoretical framework advocated by Nisbett and his colleagues (e.g., Nisbett and Masuda, 2003), this paper examines whether East Asians' context sensitivity is stronger than that of North Americans, even when the contextual information is the facial expressions of actual models. Whereas the independent/analytic understanding of the world is dominant in North American cultures, people in East Asian cultures are more likely to show sensitivity to multiple others' emotion in a given situation, and the contexts where the complex interpersonal activities are taking place. Another line of discourse in cultural psychology suggests that cultural variation in the sensitivity to context should be particularly intensified in social contexts, because East Asians and North Americans have qualitatively different perceptions of the role of social others (Markus and Kitayama, 1991; Kitayama and Markus, 1999). In fact, previous findings indicate that the types of stimuli have different impacts on people across cultures. That is, North Americans tend to regard an individual as a distinct agent whose emotions are socially disengaged and whose state of mind is a strictly personal phenomenon (Markus and Kitayama, 1994, 2010; Mesquita and Markus, 2004; Mesquita and Leu, 2007). Given North Americans' agentic understanding of emotions, the presence of social others' emotions should be less important for North Americans' interpretation of the target model's facial emotions. That is, North Americans would be likely to consider emotions as manifestations of private states impervious to the emotions of others. By contrast, East Asians tend to share the cultural belief that individuals' emotional states are strongly tied with those of social others who are significant in their lives. Thus the presence of social others' emotions would be easily merged into East Asians' interpretation of the target model's facial emotions, whereas such an effect would be less pronounced among North Americans.

In the preliminary investigation, we asked participants to watch a series of cartoon images in which a protagonist, who showed a specific facial expression, stood out from people in the background, who also showed specific facial expressions. Participants were asked to judge the protagonist's facial expression. Here we manipulated the combination of emotional expressions of the center model and background models: congruent images (e.g., happy center and happy backgrounds) vs. incongruent images (e.g., happy center and sad backgrounds). The results indeed showed partial evidence that surrounding figures' emotions strongly affected Japanese participants' judgment of target figure's emotions, while such manipulation had little effect on European Americans' judgment (Masuda et al., 2008b). The findings suggest a systematic variation in the affective context effect between Japanese and European Americans.

However, it is only recently that researchers have begun to test the generalizability of the findings to real emotion faces, to different cultural groups, and to different age groups (e.g., Ko et al., 2011). In addition, the number of studies investigating the relationship between behavioral patterns (cognitive judgment) and psycho-physiological patterns is limited (e.g., Chua et al., 2005). Furthermore, there are several methodological weaknesses in our preliminary investigation (Masuda et al., 2008b). For example, Masuda et al. (2008b) used cartoon images as the experimental stimuli. It is important to replicate the findings using photographs of real faces, which provide a closer representation of how people process information in everyday interpersonal relationships. In addition, familiarity with cartoon faces may vary culturally, and this factor should be controlled. Second, Masuda et al. intentionally made the target model large so that participants could easily identify the target of judgment. However, such a manipulation might have indirectly conveyed the message to North Americans that they should focus only on the target face during the task. To reduce this effect, the target model's saliency needs to be attenuated. Third, the amount of presentation time in Masuda et al. was not well controlled. As a result, there remains a possibility that North Americans quickly made a judgment by focusing only on the target model's face, whereas East Asians allocated enough time for their judgment by alternating their attention from the target model's face to surrounding others' faces. To control the above confounds, the current study examined whether a similar cultural variation in the effect of surrounding emotions on judgment is observable even when participants judge real facial expressions.

We also maintain that this investigation has an important implication. Recent findings in culture and neuroscience suggest that there are substantial cultural variations in neural activities in visual perception (Gutchess et al., 2006; Goh et al., 2007; Hedden et al., 2008; Lewis et al., 2008). Furthermore, researchers report that there are cultural variations in the increased magnitude of the N400 response associated with incidental or incongruent events (Goto et al., 2010; Na and Kitayama, 2011). Our aim in conducting this research is to produce a cross-culturally usable set of stimuli to further advance the research on culture and neuroscience.

We created 60 images, each of which consisted of one center model and four background models, while manipulating the combination of emotional expressions of the center model and background models: congruent images (e.g., happy center and happy backgrounds) vs. incongruent images (e.g., happy center and sad backgrounds). To test the robustness of the effect of culture, we also reduced the saliency of the protagonist, so that his/her size was now identical to that of the surrounding others. Third, we had all participants observe the stimulus image for a full 10 s, so they would have an equal amount of time to allocate their attention to the surrounding others. Finally, in addition to European Canadians as a representative group of North Americans, and Japanese as a representative group of East Asians, we collected data from Asian Canadians and Asian international students in Canada, which allowed us to test whether the pattern of attention acquired through experience is malleable rather than static. We hypothesized that (1) the degree of context effect would be strongest for the Japanese data, and weakest for the European Canadian data, and (2) Asian Canadian data and Asian international data would fall between these two extremes, but each would also differ from the other.

## Method

### Participants

Forty-four European Canadian students (30 females, 14 males, age *M* = 18.72, SD = 1.47); 44 Asian Canadian students (33 females, 11 males, age *M* = 19.55, SD =3.57); and 34 Asian international students (24 females, 10 males; age *M* = 22.06, SD = 5.05) were recruited at University of Alberta, Canada. Forty-four Japanese students (27 females, 17 males, age *M* = 18.63, SD = 0.69) were recruited at Hokkaido University, Japan. European Canadians, Asian Canadians, and Asian international students were recruited from the University of Alberta psychology participation pool on the basis of their ethnic backgrounds and citizenship. Japanese students were recruited through the Hokkaido University research participation system. European Canadians, Asian Canadians, and half of the Asian international students received credits to fulfill a course requirement; Japanese students and the rest of the Asian international students received monetary compensation of 10 Canadian dollars for their participation.

### Materials

A pilot study was conducted to ensure that all models' facial expressions were interpreted as intended and had a similar meaning across cultures when presented without any background. First, we took portraits of 20 student models^1^. These models imitated Ekman and Friesen's (1975) images of happy, sad, and neutral expressions. In addition to the neutral expressions, we asked the models to produce both extreme and moderate versions of happy and sad facial expressions (a total of five expressions per model). Based on the clarity of their facial expressions, we selected the portraits of six Caucasian models (3 females and 3 males) and six Japanese models (3 females and 3 males). Then, 42 European Canadian students (25 females and 17 males) at University of Alberta, and 21 Japanese students (16 females and 5 males) at Hokkaido University judged the 12 models' intensity of happiness and sadness based on their facial expression on a 10-point Likert scale ranging from 0 (*not at all*) to 9 (*extremely*). Participants' judgments of each figure's facial expressions indicated that overall these facial expressions were clearly and equally understood by both Canadians and Japanese^2^; thus, we confirmed that there were no effects of culture on the participants' judgments of the single models' emotional expression without backgrounds. Using these models, we created 60 images in the Photoshop 7. Each image consisted of one center model (target) and four background models.

### Procedure

After signing the consent form, participants were asked to sit in front of the eye tracker (Tobii 1750) and were told that their overall task was to judge the central persons' emotions based on their facial expressions. All participants in Canada (European Canadians, Asian Canadians, International students) received instructions in English, and Japanese participant received instructions in Japanese. To maintain the equivalency in translation, we applied the procedure used by Masuda et al. (2008b), in which the word “*feeling*” was translated into “*kimochi*,” and the word “*emotion*” was translated into “*kanjo*.”

The participants sat on an adjustable chair, and placed their chins on a chin rest to standardize the distance (30 cm) between the monitor and their faces. The 60 stimuli were presented in two different orders (1–60 or 60–1) using Clearview 2.5.1 software, with the Tobii 1750 eye tracker on a 17-inch (43 cm) monitor. The 50 Hz eye tracker allows us to measure participant's eye movement every 20 ms. Based on the threshold criteria used by Masuda et al. (2008a), we define the fixation threshold to be the attention held within a circle 20 pixels in diameter for two consecutive measurement units (40 ms). Saccadic eye movement below this threshold was not measured.

**Figure 1:**
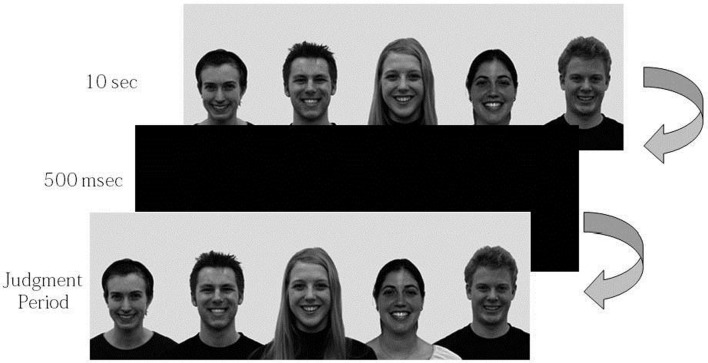
**Experimental procedure**.

Participants were first asked to observe each image for 10 s. During this process, the participants' patterns of eye-movement were measured by the eye tracker. After a 500 msec interval, they were presented with the same image again and were asked to state out loud their judgments of each central model's happiness and sadness respectively using the 10-point Likert scale ranging from 0 (*not at all*) to 9 (*extremely*) (see Figure 1). For each central model, participants were asked to provide the happiness rating first and the sadness rating second, so that the experimenter could easily record the evaluations. At the end of the experiment, participants were asked (1) whether they noticed that the emotional expression of the background figures changed, and (2) whether the background figures' emotional expressions influenced their judgments. They were then asked to fill out the demographic questionnaires and were debriefed.

## Results

### Manipulation check

The majority of European Canadians (97.73%), Asian Canadians (93.18%), Asian international students (96.97%) and Japanese (97.73%) said that they noticed changes in the expression of the background figures, and the cultural differences did not reach significance, χ^2^ (3, 166) = 1.795, *p* = 0.616. This can be interpreted as a check that the manipulation was clearly observed in all groups.

### Subjective perception of the effect of context

Our first hypothesis was that in judging the intensity of the central person's facial expressions, Japanese participants would be more likely than their North American counterparts to be influenced by the emotions of the other people. When we asked the participants whether changes in the background figures' emotional expressions influenced their judgments, we found that 59.09% of European Canadians, 59.09% of Asian Canadians, 79.41% of Asian international students, and 86.36% of Japanese stated that their judgment was indeed influenced by the surrounding others. There was a cultural difference, χ^2^ (3, 166) = 12.13, *p* = 0.007.

### Judgment data

**Figure 2:**
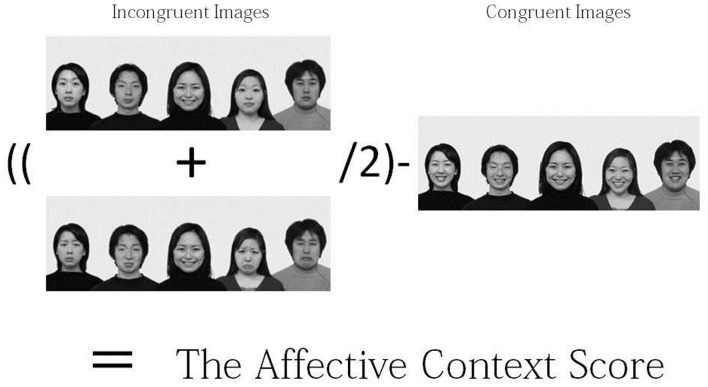
**Formula used for computing the affective context score**.

**Figure 3:**
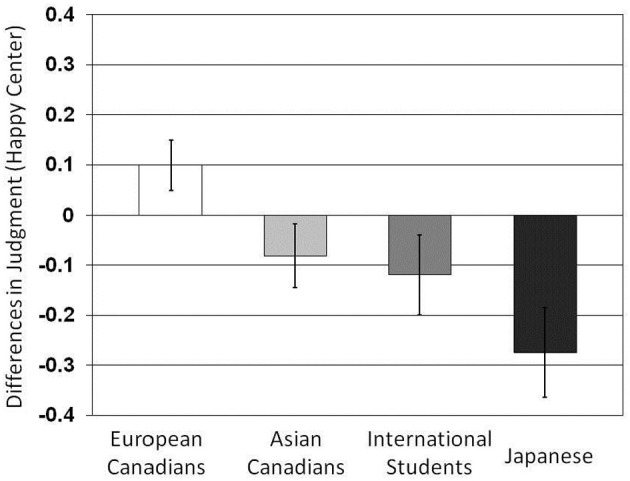
**Comparison of affective context scores for the four cultural groups**.

To examine participants' judgment styles, we collapsed the factors of models' gender and ethnicity, as well as types of target emotion, and subtracted the average judgment scores of congruent images [e.g., (happy-center and happy-background images)] from those of incongruent background images (e.g., [(happy-center and sad-background images) + (happy-center and neutral-background images)]/2; see Figure 2)^3^. A One-Way ANOVA was applied to the difference values. The results indicated that there was a main effect of culture, *F*_(1, 166)_ = 4.53, *p* = 0.004, η^2^ = 0.077. The results suggest that the more the participants were exposed to the East Asian cultural worldview, the more their judgments were influenced by changes in background figures' facial expression. In fact, the results of multiple *t*-tests revealed that Japanese (*M* = −0.47) were more likely than European Canadians (*M* = 0.01) and Asian Canadians (*M* = −0.19) to be influenced the affective contexts, *t*_(166)_ = 3.73, *p* < 0.01; *t*_(166)_ = 2.17, *p* < 0.05, respectively. The Japanese score was marginally different from that of Asian international students (*M* = −0.02), *t*_(166)_ = 1.96, *p* < 0.10. The Asian Canadian and Asian international student scores were only marginally different from the European Canadian score, *t*_(166)_ = 1.55, *p* < 0.20; *t*_(166)_ = 1.52, *p* < 0.20. When we tested whether the score of each cultural group significantly deviated from zero, the results indicated that the scores of Japanese participants, Asian international students, and Asian Canadians were all significantly different from zero, *t*_(44)_ = 3.06, *p* < 0.01. *t*_(34)_ = 2.40, *p* < 0.03; *t*_(44)_ = 2.27, *p* < 0.03, respectively. However, significance was not observed for European Canadians' score, *t*_(44)_ < 1, ns (see Figure 3).

### Eye tracking data

**Figure 4:**
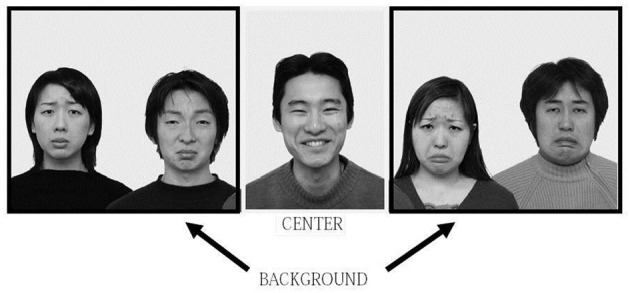
**Example of the division between the center area and the background area in an experimental image.** The total number of fixations and fixation duration for each of the two areas were recorded separately.

We again collapsed the factors of models' gender and ethnicity as well as the types of target emotion and further analyzed the eye tracking data during the first 10 s of observation. We divided the image into two areas: the center area and the background area. The number of fixations and sums of fixation durations falling in each area were measured (see Figure 4).

#### Number of fixations

**Figure 5:**
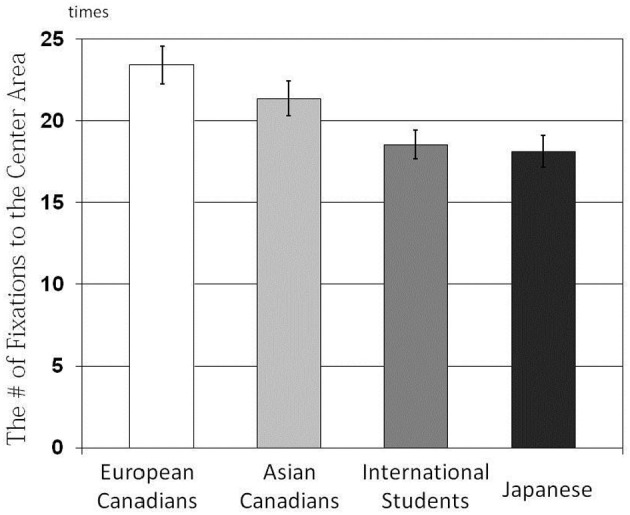
**Comparison of the number of fixations to the Center Area for the four cultural groups**.

**Figure 6:**
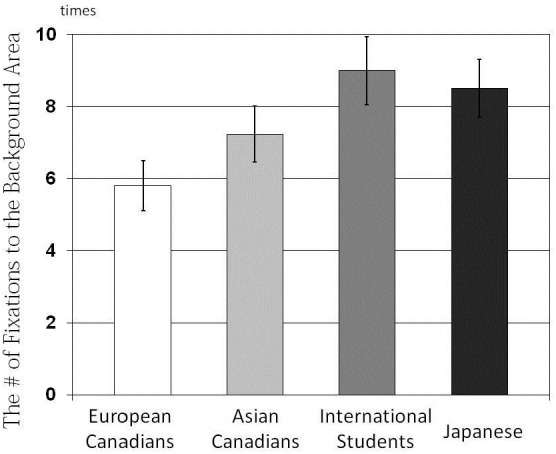
**Comparison of the number of fixations to the Background Area for the four cultural groups**.

The results of a One-Way ANOVA applied to the number of fixations indicated that there were main effects of culture on the number of fixations, *F*_(3, 162)_ = 5.88, *p* < 0.001, η^2^ = 0.098 for the Center Area (Figure 5); *F*_(3, 162)_ = 3.14, *p* < 0.03, η^2^ = 0.055 for the Background Area (Figure 6). The results of multiple *t-tests* revealed that European Canadians (*M* = 23.42) and Asian Canadians (*M* = 21.37) were more likely than their Japanese counterparts (*M* = 18.11) to allocate their attention to the Center Area, *t*_(166)_ = 3.72, *p* < 0.001, *t*_(166)_ = 2.29, *p* < 0.05, respectively. In addition, European Canadians were more likely than Asian international students to allocate their attention to the center area, *t*_(166)_ = 3.19, *p* < 0.01. Furthermore, Japanese (*M* = 8.51) and Asian international students (*M* = 9.00) were more likely than European Canadians (*M* = 5.80) to allocate their attention to the background area, *t*_(166)_ = 2.46, *p* < 0.01, *t*_(166)_ = 2.71, *p* < 0.01, respectively.

#### Sums of fixation durations

**Figure 7:**
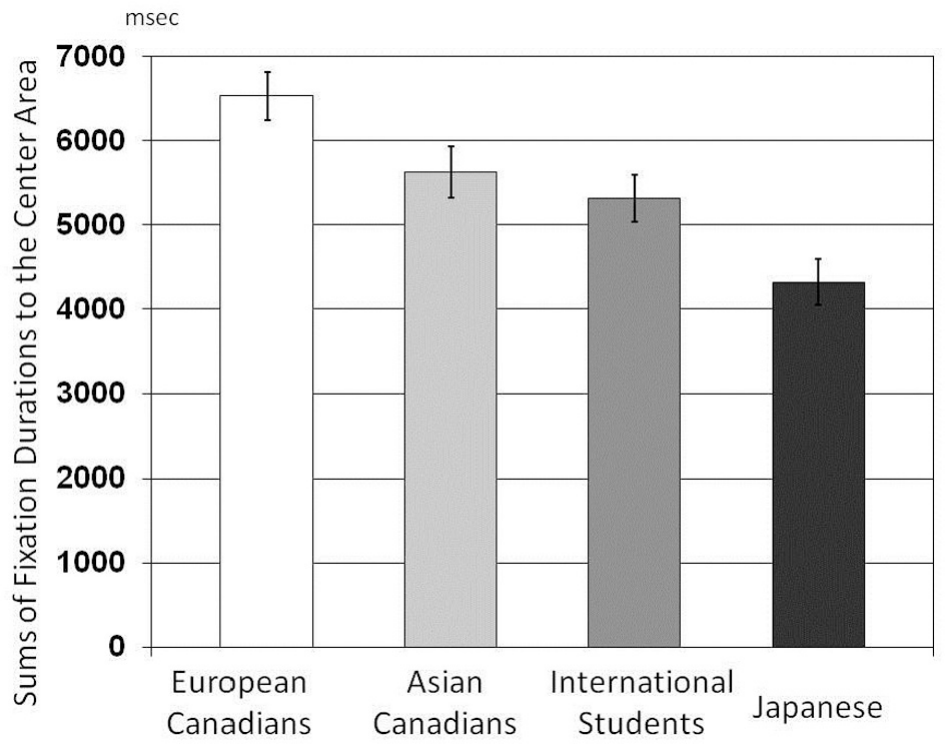
**Comparison of sums of fixation durations to the Center Area for the four cultural groups**.

**Figure 8:**
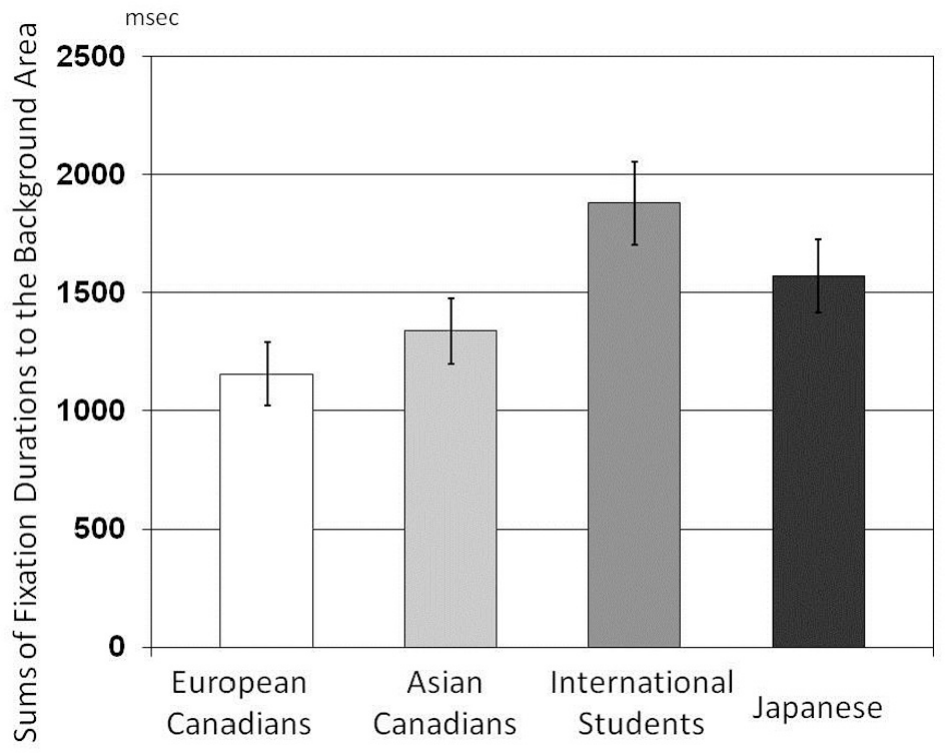
**Comparison of sums of fixation durations to the Background Area for the four cultural groups**.

The results of a One-Way ANOVA applied to the number of fixations indicated that there were main effects of culture on sums of fixation durations, *F*_(3, 162)_ = 10.73, *p* < 0.001, η^2^ = 0.166 for the Center Area (Figure 7); *F*_(3, 162)_ = 4.03. *p* = 0.008, η^2^ = 0.069 for the Background Area (Figure 8). The results of multiple *t*-tests for the Center Area revealed that European Canadians (*M* = 6533.89) were more likely than Japanese (*M* = 4322.46), Asian international students (*M* = 5314.56) and Asian Canadians (*M* = 5626.61) to allocate their attention to the center area, *t*_(166)_ = 5.62, *p* < 0.001, *t*_(166)_ = 2.89, *p* < 0.01, *t*_(166)_ = 2.31, *p* < 0.05, respectively. And, Asian Canadians and Asian international students were more likely than and Japanese to allocate their attention to the center area, *t*_(166)_ = 3.31, *p* < 0.01, *t*_(166)_ = 2.35, *p* < 0.05, respectively. Furthermore, Japanese (*M* = 1568.19) and Asian international students (*M* = 1878.20) were more likely than European Canadians (*M* = 1154.17) to allocate their attention to the background area, *t*_(166)_ = 2.02, *p* < 0.05, *t*_(166)_ = 3.29, *p* < 0.01, respectively. Asian international students were more likely than Asian Canadians to allocate their attention to the background area, *t*_(166)_ = 2.46, *p* < 0.02.

In sum, the results of eye tracking data analyses suggest that (1) Japanese attended more than their European Canadian counterparts to background figures; (2) Canadians, especially European Canadians, were more likely than Japanese to attend to the central figure; (3) Asian international students in Canada were more likely than European Canadians to allocate their attention to the background figures; and (4) Asian Canadians' eye movement data fell between the two extreme groups (European Canadians and Japanese). This suggests that the emotional context effect of the Asian Canadian and Asian international student data, although weaker than that of the Japanese data, still display the patterns of attention dominant in East Asia. Furthermore, the results indicated that participants' attention to background positively correlated with their judgment styles. The more a person paid attention to the background (as indicated by increased number of fixations and longer fixation duration), the larger the discrepancy in judgment between congruent and incongruent images, *r*_(166)_ = −0.19, *p* < 0.02 for number of fixations, *r*_(166)_ = −0.16, *p* < 0.05 for fixation duration.

This suggests that the cultural variation in emotional context effect on judgment is not grounded in superficial differences in judgment patterns but heavily grounded in participants' voluntary eye movement patterns.

## General discussion

The findings suggest that although the influence of some types of affective cues in context is observable across cultures, affective contexts used in the current study had the greatest influence on Japanese participants' judgment and the least influence on European Canadians' judgment. We maintain that the cultural variation in affective context effect observed in this study is due to differences in worldview shared by the respective cultural communities. For East Asians, the world is complex and everything is interrelated. Therefore, East Asians epistemologically apply a holistic strategy to capture the scenes, paying attention not only to the focal information but also to surrounding information that might be considered peripheral by North Americans. East Asians' interdependent tendency also facilitates the holistic pattern of attention. The results suggest that, instead of assuming that one's facial expressions are generated from his or her inner feelings, East Asians assume that facial expressions are a product of complex interpersonal relationships. Therefore, for East Asians, it is informative to attend to surrounding others' facial expressions and incorporate them into their judgment of the target model's facial expression. By contrast, North Americans share a worldview in which the world consists of discrete things that are independent from each other. Therefore, North Americans epistemologically apply an analytic strategy to capture the scenes, detecting focal faces in the scenes while differentiating them from peripheral information. North Americans' independent tendency also facilitates the analytic pattern of attention. The results suggest that North Americans assume that one's facial expression is generated from the person's inner feelings. Therefore, for North Americans, it is informative to focus only on the target agent to be assessed.

### Limitations

There are some limitations in the current studies. First, because of the technological constraints, we could not capture participants' saccadic eye movement below the threshold (40 ms and 20 pixels). However, it is possible that there are systematic cultural variations in participants' saccade. Although it is beyond the scope of the current research, it will be worthwhile to investigate this aspect using a more advanced eye-tracking device. Second, although the forced attention method used in this study overcame the shortcomings of previous work (Masuda et al., 2008b), it is still possible that there are substantial cultural variations in participants' judgment speed. North Americans might make a judgment faster than Japanese while viewing images for 10 s. Therefore it is advisable to test, in a future study, whether Japanese are influenced by changes in background when they view the image for a very short period of time, for example, shorter than that of North Americans' fastest judgment time. In fact, the findings of Masuda et al. (2008b) partially suggested this possibility. That is, Japanese participants' attention started to deviate from the center after 1 s. However, in combination with the current experimental design—forcing North Americans to view images much longer than their regular judgment speed—an experimental design that uses a shorter viewing time will further elucidate the relationship between culture, judgment speed, and the context effect.

### Implications

The current findings address a variety of research questions which future research needs to investigate. First, as aforementioned, current findings in neuroscience indicate that substantial cultural variations in the increased magnitude of the N400 response associated with incidental and incongruent pieces of information (Goto et al., 2010; Na and Kitayama, 2011). On the basis of these neuroscientific findings that indicate the cultural specificity of the N400 response (e.g., Goto et al., 2010), we assume that East Asians will be more likely than North Americans to have an increase in the magnitude of the N400 response when they observe an image in which the target facial expression is incongruent with that of the background figures. Future neuroscientific research should investigate this possibility so as to further elucidate cultural variations in the mechanism of information processing.

Second, it is advisable to further discuss the issue from the gerontological perspective. For example, Ko et al. (2011) found that cultural variation in context sensitivity was observed only among young adults and not among older adults. They interpreted this to mean that the ability to incorporate background information declines with age. However, the background images and foreground images used by Ko et al. are visually dissociated from each other (e.g., a target human face detached from the body was placed against a picture of a snake, the texture and resolution of which were quite different from those of the target face), which increases the difficulty of perceptually integrating them. The stimuli used in the current study, however, use the same texture and resolution for both the background and foreground images, which may allow participants to easily integrate these pieces of information. We suggest that our stimuli be used in future research to examine whether the cultural variation in context sensitivity is observable even in older adults.

Third, the issue of malleability of attention need to be investigated. Is one's pattern of attention static? We maintain that this is unlikely. From an early age, a person learns the dominant worldview of a given society through interaction with people in that culture (Nisbett, 2003; Duffy and Kitayama, 2010), and if that person moves to a new culture, his or her way of thought gradually blends the worldview of the host culture with that of the original culture. Much cross-cultural research that involves both monocultural and bicultural participants has reported such a blending pattern. That is, the bicultural group's performance falls somewhere between that of two monocultural groups (Kitayama et al., 1997; Heine and Lehman, 2004; Tsai et al., 2004; Heine and Hamamura, 2007; Cheung et al., 2011). These findings suggest that patterns of behavior are not static, but blend malleably into new circumstances. Some data indeed indicate that one's patterns of attention are learned (Kitayama et al., 2003; Duffy et al., 2009) and malleable (Miyamoto et al., 2006). The current paper demonstrated that the affective context effect observed in Asian Canadians' and Asian international students' data was somewhat weaker than the Japanese data, and somewhat stronger than the European data. In fact, although the affective context effect was weaker for Asian Canadians and international students than for their Japanese counterparts, both judgment data and most of eye tracking data show that Asian Canadians' and international students' patterns of judgment and patterns of attention reside between those of the two extremes (European Canadians and Japanese).

Several attempts have been initiated to further test the compatibility with previous findings and to articulate the cross-cultural variations and similarities of the affective context effect. In this line of studies, researchers have attempted to determine the conditions in which the affective context effect is observed, that is, what types of judgment tasks and what types of affective stimuli accentuate or attenuate the affective context effect. For example, Ito and Masuda (submitted) used stimuli in which the target model's face was presented against either affectively congruent or incongruent scenes (e.g., the target's happy facial expression against a beautiful beach as background, vs. the same facial expression shown against a dirty toilet). Results of the rating task indicated that even North Americans experienced the affective context effect. Using the same stimuli, Ito et al. (2012) asked participants to simply make a quick categorization of the valence (positive vs. negative) of the target facial expression, and again demonstrated that that European Canadians and Japanese equally responded faster when targets' facial emotions were affectively congruent with contextual information than when they were incongruent. The findings of these two studies indicated that affective context effects are indeed observable across cultures, but that cultural variation in the affective context effect is accentuated only when the task involves deliberate rating of the target models' facial expression and when the target agents are surrounded by social others. We maintain that this line of investigation is important for future research because it will contribute to an understanding of cultural variations and universals in neural substrates of psychological processes. We also maintain that this research paradigm will shed light on individual differences in sensitivity to social others. For example, it will be informative to examine what types of personality characteristics and clinical attributes are associated with stronger or weaker affective context effects, and what causes these variations in judgment (e.g., Risko et al., 2012).

## Summary

In sum, while reducing potential confounding variables in Masuda et al. (2008b), the current findings suggest that cultural variation in emotion judgment is substantial even when real facial expressions are used. This finding has many implications for research on emotion, cognition, and neuroscience. Current findings in neuroscience indicate that substantial cultural variations in the increased magnitude of the N400 response associated with incidental and incongruent pieces of information (Goto et al., 2010; Na and Kitayama, 2011). On the basis of these findings, we assume that East Asians will be more likely than North Americans to have an increase in the magnitude of the N400 response when they observe an image in which the target facial expression is incongruent with that of the background figures. Future neuroscientific research should investigate this possibility so as to further elucidate cultural variations in the mechanism of information processing. In addition, the current set of real face stimuli will contribute to further advance research on emotions and corresponding facial expressions. Because many researchers have focused on the consistency between emotion and facial expression, they tended to put less importance on external factors such as the context effect on emotion judgment (e.g., Ekman and Friesen, 1975). However, various researchers have discussed the importance of context for one's emotion judgment (e.g., Carroll and Russell, 1996; Hietanen et al., 2007). Along with these findings, the current paper will facilitate the discussion regarding the malleability of context attention across age (e.g., Miyamoto et al., 2006; Duffy et al., 2009; Ko et al., 2011), and the magnitude of the context effect across situations (e.g., Ito et al., 2012).

### Conflict of interest statement

The authors declare that the research was conducted in the absence of any commercial or financial relationships that could be construed as a potential conflict of interest.

## Footnotes

^1^All the models agreed to let the researchers use their portraits for research purposes. The models were not professional models but were students who were recruited through a school flyer.

^2^Canadian and Japanese participants' judgment was statistically different regarding the neutral expression of only one model, *t*_(59)_ = 2.07, *p* = 0.043. Other than that, there was no cultural variation in judgment between Canadian and Japanese participants.

^3^We also carried out a full factorial model of the types of target emotion, model's gender, and ethnicity. The results indicated an effect of types of target emotion. In general, the differences in score between congruent facial expressions and incongruent expressions is larger when participants judged targets' happy faces than when they judged targets' sad faces, *F*_(1, 162)_ = 10.77, *p* = 0.001, η^2^ = 0.062. However, there were no effects of model's gender or models' ethnicity, *F*_(1, 162)_ = 1.55, ns, *F* < 1, respectively. In addition, no interaction terms produced statistically significant results. We therefore are confident regarding the equivalence of image quality used in this study.
